# Behavioural and physiological responses to salinization and air exposure during the ontogeny of a freshwater South American snail

**DOI:** 10.1093/conphys/coac089

**Published:** 2023-01-28

**Authors:** R Barrios-Figueroa, M A Urbina

**Affiliations:** Departamento de Zoología, Facultad de Ciencias Naturales y Oceanográficas, Universidad de Concepción, Chile; Doctorado en Sistemática y Biodiversidad, Facultad de Ciencias Naturales y Oceanográficas, Universidad de Concepción, Concepción, 4030000, Chile; Departamento de Zoología, Facultad de Ciencias Naturales y Oceanográficas, Universidad de Concepción, Chile; Instituto Milenio de Oceanografía (IMO), Universidad de Concepción, PO Box 1313, Concepción, 4030000, Chile

**Keywords:** salinization, salinity tolerance, molluscs, freshwater snail, Estuaries

## Abstract

Salinization is of global concern, threatening freshwater biodiversity. Salinity tolerance is highly variable and therefore needs to be evaluated on a species-specific basis. An estuarine population of *Chilina dombeiana,* a freshwater gastropod endemic to Chile and classified as vulnerable, has been recently found in the Biobío River’s mouth, suggesting some degree of tolerance to brackish waters. This study evaluated the survival, behaviour (medium preference) and physiology of *C. dombeiana* when exposed to salinities higher than freshwater, thus elucidating the potential mechanisms used to survive salinization. *Chilina dombeiana* belongs to the Pulmonate group;, so we evaluated oxygen uptake in air and water, aiming to evaluate emersion as a potential avoidance response to a progressive salinity increase. Complete embryo development was observed for salinities ≤ 16 PSU (practical salinity units) but hatching rates above 50% were only achieved in freshwater (0 PSU). It was also found that salinity had stage-specific effects during embryonic development. In adults, acute exposure to brackish water (12 PSU) caused a decrease in oxygen consumption (compared to freshwater), in the ammonium excretion rates and in the percentage of muscular water content. Although *C. dombeiana* was able to take up oxygen in both mediums, survival in air decreased over time (days), which correlates with the behavioural preference to remain submerged, even at elevated salinities. Considering the survival of adults and embryos decreased as salinity increased and the lack of an avoidance behaviour or a physiological ability to maintain homeostasis at salinities higher than freshwater, our results suggest this snail could be adversely affected by salinization in the long term. Furthermore, given the ability of *C. dombeiana* to uptake oxygen in both mediums, it should be considered as a facultative air breather snail, rather than a strictly aquatic species.

## Teaser

Salinization is a global concern, threatening freshwater species and biodiversity. We studied the effects of salinization on the behavioral and physiological responses of a south American freshwater snail. Embryonic development in salinities other than 0 PSU (Practical Salinity Units) were possible, but at expense of lower survival rates. However, adults acutely tolerated up to 12 PSU without any detrimental effects.

## Introduction

Stream salinization is an increasingly frequent phenomenon that threatens freshwater organisms and global biodiversity. It can be caused by agricultural land use, discharge of mining effluents, aquaculture effluents, hydraulic fracking and sea intrusions caused by global warming. Detrimental impacts of salinization have been reported from organism level to whole ecosystem damage (Schäfer *et al.*, 2012; Cañedo-Argüelles *et al.*, 2013; Cañedo-Argüelles *et al.*, 2016; Olson, 2019).

Mean sea levels have increased by ~ 20 cm over the 20th century (Yu *et al.*, 2003), causing saline waters to intrude upstream into previously freshwater environments (Poff *et al.*, 2002). These intrusions not only cause salinization (Hilton *et al.*, 2008; Bhuiyan and Dutta, 2012) but also lead to changes in stratification and estuarine circulation (Hong and Shen, 2012). The rate of rising sea level has reached unprecedent magnitudes in recent years (4.4 mm year^−1^) due to the accelerated climate change (IPCC, 2021).

Salinity has long been considered as one of the most important environmental factors in aquatic ecosystems, as it limits species distribution (Kinne, 1971) and affects organism physiology (Cañedo-Argüelles *et al.*, 2019) and development (Kefford *et al.*, 2004; Kefford *et al.*, 2007; Zukowski and Walker, 2009), thus shaping the abundance of aquatic invertebrates (De Silva and Perera, 1976; Eckert *et al.*, 1989). It has been shown that salinity may have differing effects during different stages of species ontogeny and that ignoring earlier stages and assessing its effects only on adults may result in inaccurate estimates of true tolerances (Kefford *et al.*, 2004; Kefford *et al.*, 2007). In early life stages of freshwater snails, such as when embryos are inside their eggs, an increase in salinity has been reported to cause a decrease in hatching and growth rates of *Glyptophysa gibbosa*, *G. aliciae* and *Glacidorbis* species (Kefford *et al.*, 2007). In *Physa acuta* embryos, for example, a study has shown that that all embryos hatched at salinities below 1.67 PSU (practical salinity units), while a significant reduction in hatching was observed at 7.22 PSU, followed by an absolute impairment of hatching at 8.22 PSU (Kefford *et al.*, 2004). One member of the Lymnacidae group (unidentified specie) showed hatching rates above 90% at salinities lower than 5.63 PSU, but at 8.72 PSU no embryo left the egg (Kefford *et al.*, 2004). Similar occurrences have been observed for the limpet *Burnupia stenochorias*, which presented hatching rates above 94% at salinities up to 3.49 PSU, but only minor increases in salinity caused a sharp decrease in hatching (Kefford *et al.*, 2004).

The effect of salinity through snail ontogeny has also been evaluated, in *G. gibbosa* and *P. acuta*, where hatching time increased and egg survival decreased to 0% at 4.2 and 5.2 PSU, respectively, a pattern that was also found in juveniles and adults (Zukowski and Walker, 2009). In adult *P. acuta*, elevated salinities significantly reduced survival, with stark differences over small increases (Stockwell *et al.*, 2011).

Species have an optimal range of environmental salinities in which they balance ecological interactions (preys and predators) and energy expenditures even when the salinities deviate from their isosmotic point and thusly more energy must be expended on osmoregulation (Bayne, 1976). Salinization directly challenges freshwater organisms, moving them away from this balance and likely forcing them to invest more energy into osmoregulation in order to achieve a new equilibrium (Peterson-Curtis, 1997; Paganini *et al.*, 2010; Peña-Villalobos *et al.*, 2016). Freshwater organisms are immersed on a diluted medium, having internal fluids hyper concentrated with respect to the external medium and therefore continuously facing a passive loss of ions to the environment and a passive gain of water (Krogh, 1939; Smith, 1961; Marshall and Grosell, 2005). These gradient-driven passive losses are compensated through the active pumping of ions from the environment into the animal, at the expense of ATP (Adenosine triphosphate) (Salvato *et al.*, 2001), contributing to intracellular isosmotic regulation, the primary physiological mechanisms to cope with osmotic fluctuations in the external environment (Matsushima *et al.*, 1987). Because maintaining internal osmolality involves the action of several ATP-fuelled transporters, it is energetically expensive (Boeuf and Payan, 2001). Therefore, suboptimal salinities affect several physiological processes such as respiration, excretion, filtration rates and growth rates (Pourmozaffa *et al.*, 2019). At the end of protein metabolism, one of the mechanisms aquatic animals use to excrete nitrogenous waste products (ammonium: NH_4_^+^) is through an indirect coupling with the absorption of Na^+^. Considering that Na^+^ and Cl^−^ are the most abundant ions in seawater (30.65% and 55.03%, respectively) (Pilson, 1998), any changes in the external Na^+^ concentration might also affect the excretion of ammonium (Wright and Wood, 2009).

Amongst freshwater invertebrates, molluscs have diversified into various bodies of freshwater, with 17.5% (~7000) of the total gastropod species described inhabiting freshwater systems (Cuezzo, 2009; Naranjo-García and Olivera-Carrasco, 2014). Of the 73 continental water species in Chile, 30 species belong to the genus *Chilina* Gray 1828, all described as pulmonated (Valdovinos, 2006). Aquatic pulmonated snails possess both lungs and accessory gills, allowing them to capture oxygen from air and water (Zukowski and Walker, 2009). The Chilinidae genus belongs to a family classified with a conservation status of vulnerable, primarily due to a continuous decline in habitat availability and quality as a result of anthropogenic contamination (Valdovinos *et al.*, 2004; Figueroa *et al.*, 2007).

*Chilina dombeiana* (Bruguière, 1789) is a freshwater snail endemic to Chile (Jarne *et al.*, 2010), widely distributed in riverine ecosystems between 35° and 41° S latitude (Bórquez and Brante, 2017; Valdovinos, 2006). As a freshwater organism, *C. dombeiana* is vulnerable to anthropogenically driven changes on its habitat (Bórquez, 2013), such as salinization. This species presents simultaneous hermaphroditism; it is oviparous with internal fertilization and direct development. Egg masses are deposited on rocky substrates (Bórquez *et al.*, 2015), between December and March of the southern summer season (Bisol *et al.*, 1994; Bórquez, 2013). Each egg string contains a variable number of embryos (60–298 eggs); each embryo is individually encapsulated and embedded into a gelatinous matrix, where they develop through five embryonic stages over 28 days (18°C) (Bórquez *et al.*, 2015). The lack of free-swimming larval stages limits their dispersal (Bórquez and Brante, 2017) but assures that juveniles will experience similar conditions to those experienced by adults (likely suitable). Despite its traditional description as a freshwater species, an estuarine population has also been described (Bórquez *et al.*, 2015), yet the physiological adaptations this freshwater snail uses to tolerate salinities higher than freshwater have not been explored to date. 

The aim of this study was to evaluate the salinity tolerance during the whole ontogeny of *C. dombeiana*, evaluating the potential behavioural and physiological responses that allow such tolerance. We evaluated embryonic development, survival and hatching over ~ 30 days at 0, 8, 16 and 24 PSU salinities. The species preference for either aquatic or air mediums, and their ability to uptake oxygen from both mediums, was also evaluated in adults. Additionally, adults were acutely exposed to brackish water in order to evaluate their metabolism, ammonium excretion and intramuscular water content, as a proxy of their osmoregulatory capacity. As an aquatic organism, we hypothesize that *C. dombeiana* will prefer to remain submerged in the water medium where it will achieve better survival and more efficient oxygen uptake compared to air. Regarding the effects of salinity, we hypothesize exposure to brackish waters (at some threshold) will lower ammonium excretion rates and intramuscular water content but will demand an elevated oxygen consumption to fuel the higher costs related with to achieving a new equilibrium with the external medium. Given its ability to emerse, we also hypothesize *C. dombeiana* will emerse as an avoidance behaviour when facing fresh water salinization.

## Materials and Methods

All experimental procedures comply with the existing Chilean regulations and were approved by the Universidad de Concepcion ethical committee.

### Animal collection and acclimation

Adult *C. dombeiana* individuals were collected manually during low tide, south of the Biobío River mouth (Boca Sur, San Pedro de la Paz 36° 49 ‘30 0.05”S; 73° 8 ‘56 0.77′ ‘O). Snails were placed in coolers with water from the site of collection, provided with gentle aeration and transported to the Laboratorio de Fisiología Animal Comparada, at Universidad de Concepción. They were maintained in 5 L aquariums with fresh water (0.1 PSU, and equivalent to grammes per litre) and static flow, inside a temperature-controlled room set at 14 ± 0.3°C, with continuous aeration and a 12 L:12D hours light: dark cycle. Water was renewed every other day. Snail feed on the periphyton that growth on the holding tanks; therefore, they were food deprived as soon they start the acclimation for any of the following experiments.

### Experimental design

Salinities above freshwater (~ 0 PSU; dechlorinated Concepcion city tap water) and below seawater (~32 PSU, collected from Dichato bay, Marine Biology station of the University of Concepcion) were obtained by mixing both fresh and sea water. Salinity was checked by a salinity metre (ThermoFisher Orion Star™ A222).

### Embryos

#### Survival and development

Survival and development during each embryonic stage were evaluated for a total of 31 days at 0, 8, 16, 24 and 32 PSU experimental salinities, on 96-mL glass chambers. Five recently laid (within 6 hours) egg strings (replicates) from five different females were cut into portions containing 20 eggs each. This was performed under a microscope, using a scalpel. One section of egg string from each female was allocated to each of the five experimental salinities, and therefore each salinity treatment had similar genetic diversity (strings from five different females). Eggs were observed daily under an optical microscope (Olympus, magnification 10X and 40X) during the first 3 days of exposure, and then every two days until day 31. Water was changed on the days of observation. Survival and time until the next embryonic stage was recorded; embryos reaching stage VI were recorded as completing development. The stages were differentiated according to Bórquez *et al.*, 2015.

### Adults

#### Survival and preference in aquatic and aerial mediums

Before salinity exposures, survival and preference in aquatic and aerial mediums were evaluated. Survival in aquatic and air mediums was evaluated using 30 adult snails divided into 3 replicates of 10 snails each (*n* = 3) for each medium. Three replicates were randomly allocated to the aqueous treatment in 0.5 L containers, which were filled with dechlorinated water (0.106 PSU). The upper opening was secured with meshes so that the snails could not get out, and they were immersed in a larger aquarium with dechlorinated water so the snails allocated to the aquatic medium were effectively submerged. Another set of three replicates were allocated to the air exposure treatment, in similar 0.5 L containers, but lacking water. Survival was recorded every 12 hours, over 7 days.

To examine medium preference, adult snails (*n* = 20, 1.35 ± 0.80 g, 19.5 ± 3.30 mm) were individually placed in 0.25 L containers filled halfway with dechlorinated fresh water (0.106 PSU), allowing the free movement of the snails between water and air. They were observed over a period of 6 hours, registering their position with respects to water or air, every 15 minutes. Emersion was considered to be the snail was completely out of water, and medium preference was expressed as the percentage of the observations time that the snails were either in or out of water. Position was checked every 15 minutes, based on the low locomotion of snails, yet the observer was always present.

#### Behavioural avoidance response during progressive salinization

Avoidance behaviour, the choice of emerging from water into air when water salinity is increased, was evaluated in 20 adults (1.05 ± 0.47 g, 19.0 ± 2.94 mm). The snails were placed individually in 0.25 L containers, filled with 0.15 L (5 cm height) of water. The remaining 0.10 L volume (another 5 cm height) allowed the snail to emerse if desired. A perforation halfway up the container allowed sea water to be added without changing water volume (and thus height) or disturbing the animals. All snails were submerged at the beginning of the experiment in fresh water (0.1 PSU), and their position was recorded every 15 minutes during 1 hour. After this initial time in freshwater, the salinity was increased by 2 PSU every hour up to 8 PSU (salinities: 2.08, 4.05, 6.01, 8.07 PSU). After this initial hourly and progressive salinity increase, salinity was further increased to 12, 18 and 24 PSU, every 0.5 hour. The temperature was maintained constant at 14°C, inside a temperature controller room.

#### Salinity exposures, survival

Adult snails (*n* = 240, 1.09 ± 0.49 g, 18 ± 3 mm) were randomly divided into groups of 10 snails and placed 0.5-L containers at 0.1, 8, 16 and 24 PSU salinities (*n* = 6) for 34 hours. The containers were covered with mesh allowing continuous aeration while preventing snails from climbing out of their containers. They were also immersed in a larger aquarium at the desired salinity, to make sure that the snails were effectively exposed to their medium. In order to replicate their natural behaviour, they were left submerged for 10 hours (at their corresponding salinity), followed by a 2-hour emersion period, allowing them to air breath (if required). To expose the snails to air, water was withdrawn from the replicate tanks by syphoning, with minimal disturbance for the animals. Survival was checked in each replicate tank at 1, 2, 3, 5, 10, 22 and 34 hours from the beginning of the salinity exposures.

#### Physiology

Based on previous experiments, 12 PSU salinity was selected for an acute challenge, being the average between the highest salinity that achieved 100% survival (8 PSU) and the lowest causing significant mortality (16 PSU) during the 34-hour exposure. Snails were acutely exposed to either freshwater (0 PSU, *n* = 10) or brackish water (12 PSU, *n* = 10); oxygen consumption and ammonium excretion were measured before and post exposure. Intramuscular (foot) water content was measured at the end of the exposure period, by excising most of the foot muscle.

#### Oxygen consumption in water and air

Twenty snails were weighed to the nearest 0.0001 g (Precisa XB 120A) and individually placed on a respirometry glass chamber (23 mL), covered with mes and submerged at 0 PSU with constant aeration during 12 hours of acclimation. Three chambers lacking snails, for each treatment, were used as controls. At the end of this period, the glass chambers were drained and the snail was left undisturbed for 30 minutes. The respirometry chambers were then closed, measuring the initial oxygen concentration on air and sealed, and then, after 3 hours and 50 minutes of incubation, the final oxygen concentration inside the chambers was measured. Then, all chambers were refilled with water at the initial salinity. After that, the initial oxygen concentration was measured, and 1 hour later, the final concentration was measured again. The containers were drained again, left for 30 minutes and sealed to perform measurements of oxygen consumption in air for 2 hours. Initial and final partial oxygen pressures were determined by inserting a needle type optic oxygen probe into the container, connected to a FireSting oxygen metre, calibrated using a 100% saturation (aerated water) and a 0% saturation solution for each salinity (Sodium thiosulphyte). Another sensor was calibrated on air using humid air (100% air saturation) and N_2_ gas (0% air saturation). In each respirometry measurement, the chambers were hermetically sealed. Oxygen concentrations were calculated using the coefficient of oxygen dissolution for each salinity at the measured temperature.

#### Ammonium excretion and muscle water content

After oxygen consumption measurements, a 1 mL water sample was taken from each respirometry chamber (0 and 12 PSU, and their respective controls) and stored in a 1.5 mL bullet tube on ice. Snails were left to aerate individually for a further 2.5 hours, and a final 1 mL sample was taken from each respirometry chamber. The concentration of ammonium in the samples and a calibration curve (NH_4_Cl) were evaluated by the indophenol method (Ivančič and Degobbis, 1984) and adapted for microplates. Samples were run in triplicates on a microplate and read at 630 nm in BioTec plate reader (EL 800).

After the final water sample for ammonium was taken, the snails in their individual chambers were returned to bigger tanks at their respective salinities, 0 or 12 PSU (10 organisms per salinity), with continuous aeration. Snails were maintained at these salinities for further 24 hours, after which they were euthanized, and the foot muscle excised (initial weight: ~90 mg). Samples were placed in pre-weighed aluminium foils, weighed again and dried at 50°C for 4 days (WTC Binder), having constant weight. Final weight was again recorded to calculate muscle water content as the difference between wet and dry weight (Urbina *et al.*, 2013).

### Statistical analysis

All data are presented as mean ± SD. All analyses were performed in R (version i386 4.0.1). Prior to performing parametric tests, assumptions of normality and homogeneity of variance were tested using Shapiro–Wilks W and Levene’s tests. For repeated measurements, the assumption of sphericity was evaluated with the Mauchly sphericity test. Survival over the exposure time on embryos was evaluated using a Chi-square test, comparing against the 100% survival achieved by the control (0 PSU), as expected. To evaluate the effects of salinity on the total and stage duration of embryonic development, a Kruskal–Wallis test was performed to assess salinity effects within each state.

In adults, the preferences for aquatic and aerial mediums were compared by Paired *T*- test, and survival in aquatic and air mediums was evaluated by Chi-square test, as well as the survival at different salinities (comparing against the initial 100% survival of each treatment). Potential differences in the oxygen consumption rates were tested by a mixed two-way ANOVA, using medium and salinity as factors, with medium as repeated measure (same snails at both mediums). Ammonium excretion rates and intramuscular water content between experimental salinities were evaluated by a *T*- test. Significance threshold was α = 0.05.

## Results

### Embryo survival

**Figure 1:**
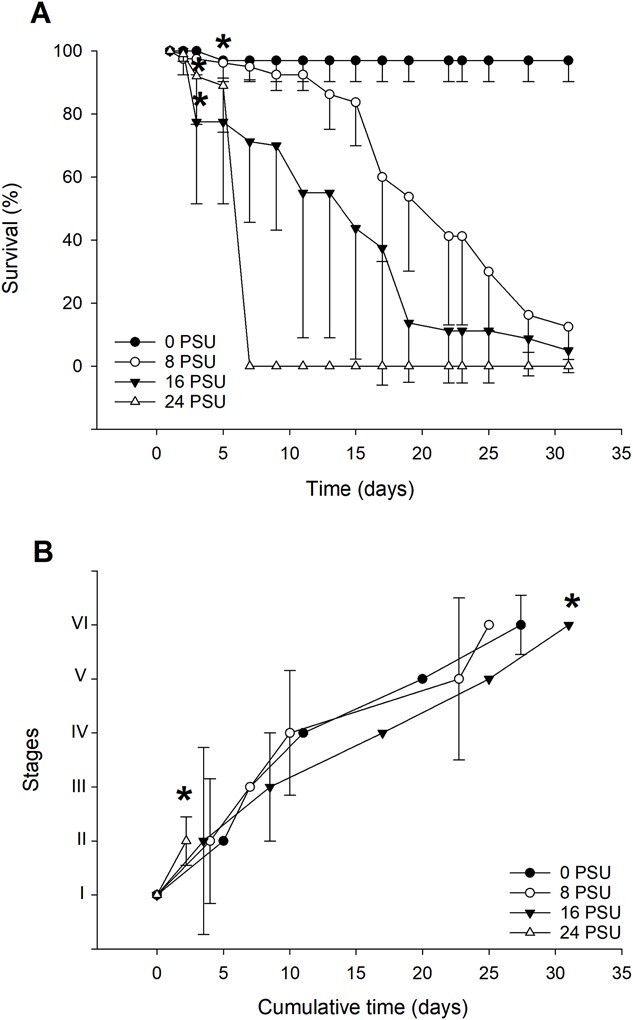
Embryonic development at different salinities (*n* = 5 per salinity, with each egg mass fragment containing 20 embryos). (A) Average survival during embryonic development at different salinities. Error bars represent standard deviation, and asterisks indicate when significant differences were achieved comparing against 0 PSU. (B) Duration of the development, total and per embryonic stage. Asterisks indicate when significant differences were achieved between treatments.

Survival was negatively affected by increases to environmental salinity, in a dose-dependent fashion (Fig. 1a). The highest salinity, 32 PSU, caused a 100% mortality within 48 hours of exposure (*P* = 2.2 × 10^−16^). Intermediate salinities of 16 and 24 PSU generated significant mortalities on day 3 (*P* = 4.77 × 10^−7^ and 0.003, respectively), while exposure to 8 PSU salinity elicited a significant mortality only after 7 days, compared to the initial 100% (*P* = 0.024).

### Embryonic development duration

Only the embryos allocated to the three lowest salinities completed stage V and hatched. Yet, because survival was not greater than 50%, development time was not evaluated at this stage. The completion of embryonic development differed between 8 and 16 PSU (obs. dif. = 7.5, critical diff. = 7.3), however, survival was lower than 50% for both treatments (Fig. 1b) Individuals in the 24 PSU treatment group (48 hours) developed, on average, faster than controls (120 hours) during stage II (obs. dif. = 9.6, critical dif. = 8.9). However, they all died after this stage. During stage III, embryos exposed to 16 PSU were delayed by 5 days, compared to controls (obs. dif. = 7.0, critical dif. = 6.9). Completion of stage IV took longer at 8 PSU compared to 16 PSU; however, treatment with 16 PSU had a lower survival.

### Adults

#### Survival and preference in aquatic and air medium

Snails presented a 100% survival in water during the exposure time, while emersion caused a significant decrease in survival. The experiment was stopped when the air exposure treatment reached 50% mortality (168 hours), but survival reached a significant drop at 144 hours, with 70% survival (air) versus 100% (water) (*P* = 0.021).

**Figure 2:**
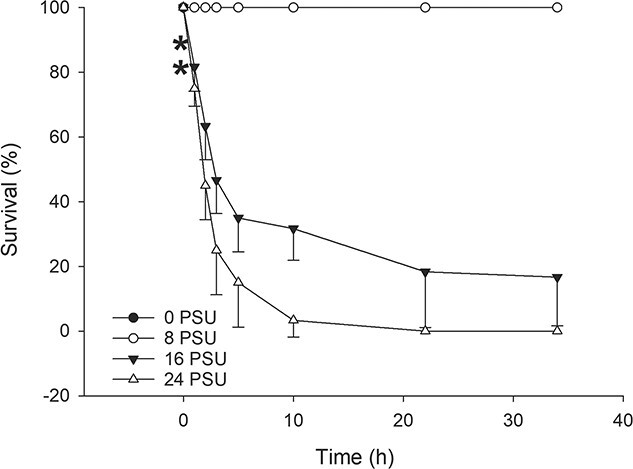
Adult preference for air and aquatic mediums, and also after an acute salinity rise (*n* = 20). (A) Number of emersions made by each snail. (B) Percentage of individuals observed in each medium. Asterisks indicate significant differences over time. (C) Percentage of snails with respect to the medium preferred during an increase in salinity over time.

When given the option of remaining submerged or to emerse, 60% of the snails remained in water during all observations. The maximum number of emersions made by one snail was three, with a mode of one (Fig. 2a). There was a significant difference in the preference between mediums, with a higher preference for water (84.37 ± 28.58) versus air (15.60 ± 28.54) (*P* = 3.39 × 10^−5^) (Fig. 2b).

#### Behavioural avoidance response during progressive salinization

Regarding behavioural responses during progressive salinization, at least on 80% of the observations the snails remained in water regardless of the progressive increases in external salinity (Fig. 2c), showing no signs of avoidance.

#### Adult survival (acute 34 hours)

**Figure 3:**
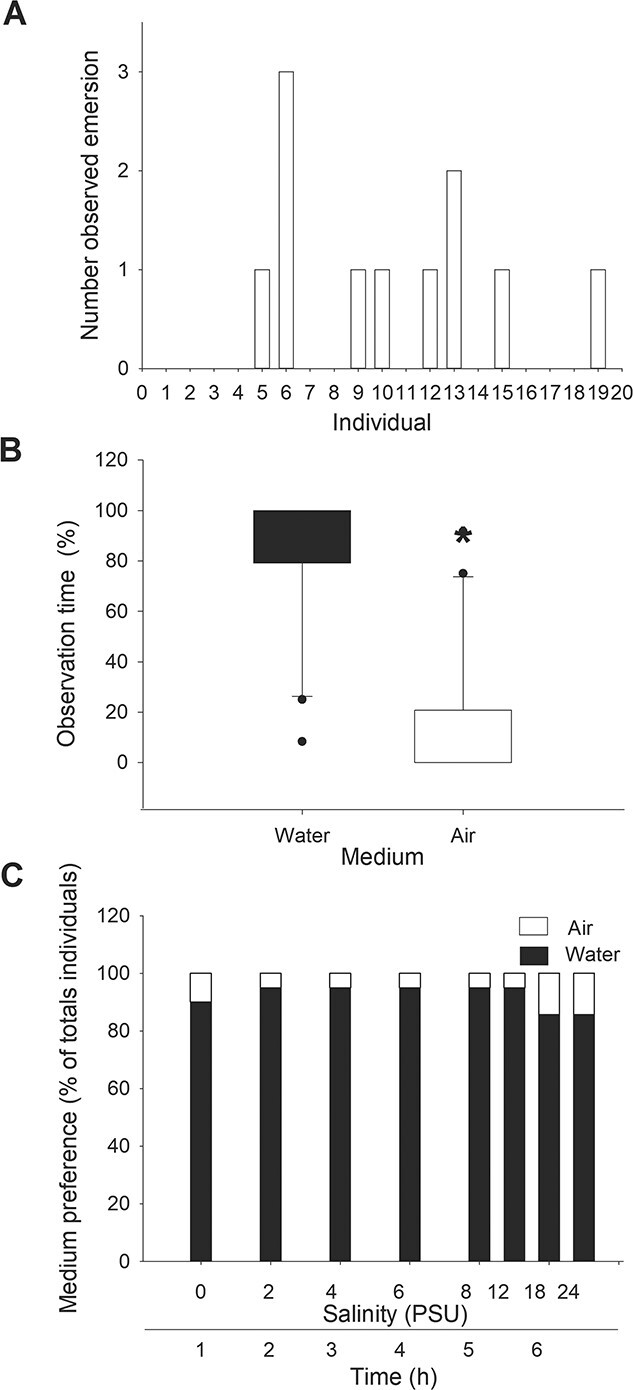
Adult survival over time, at different experimental salinities (*n* = 6 per salinity, each containing 10 snails). Error lines correspond to standard deviations, and asterisks indicated when a significant difference was reached compared to 100%

Salinity also had a negative effect on adults’ survival, with only the 8 PSU treatment not differing from the control salinity. After only 1 hour of exposure at both 16 and 24 PSU, significant differences to the groups with 100% could be observed with rates decreasing to 81% and 75%, respectively (*P* = 7.18 × 10^−6^ and 9.03 × 10^−3^). After 34 hours at 16 PSU, survival dropped further to 16%, and after 22 hours at 24 PSU, survival was at 0% (Fig. 3).

#### Oxygen consumption

**Figure 4:**
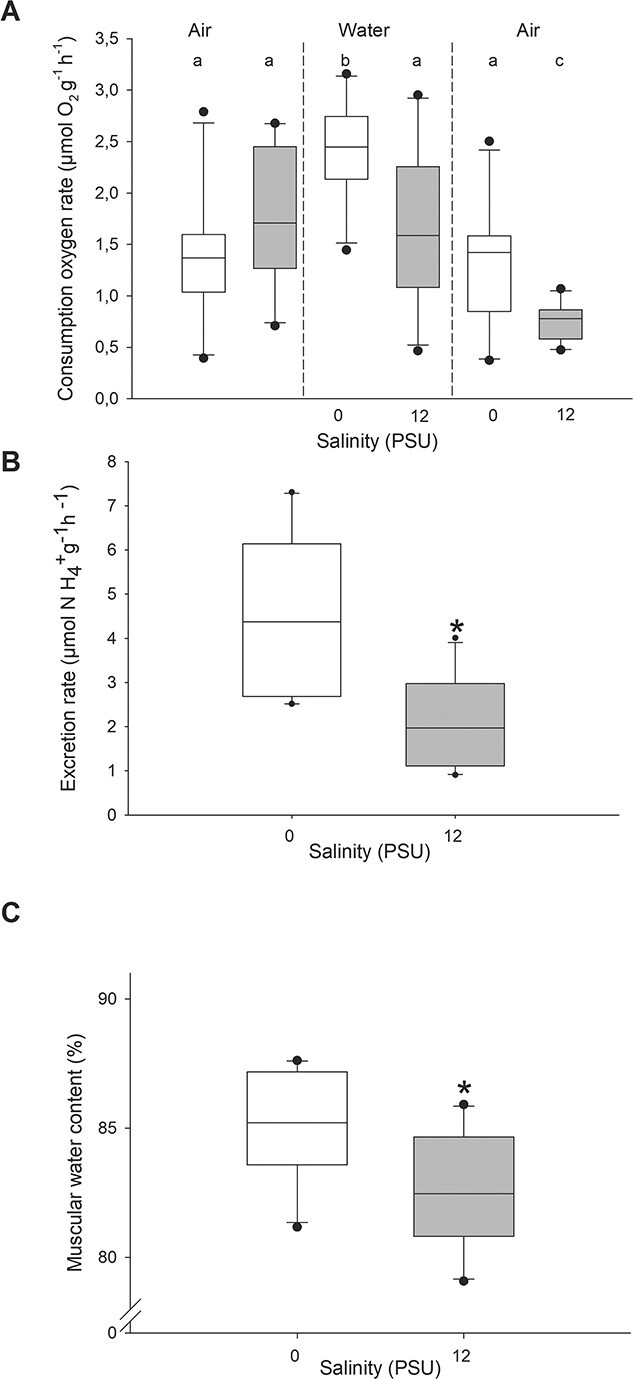
Physiological responses to an acute salinity exposure (*n* = 10 per salinity). (A) Oxygen consumption rates in control 0 and 12 PSU challenge, previous exposure, during exposure post in water and on air after exposure. The letters indicate significant differences between salinities (first lowercase), medium and pre-exposure (uppercase) and between water and air post exposure (second lowercase). (B) Ammonium excretion rates at the two salinities. C) Percentage of muscular water at the two salinities. Asterisks indicate significant differences between treatments.

Oxygen consumption was significantly reduced after exposure to brackish water. Medium (air/water) also had a significant effect, with lower rates of oxygen uptake while in air (Fig. 4a). The average oxygen consumption in freshwater was 2.44 ± 0.47 μmol O_2_ g^−1^ h^−1^, while at 12 PSU was nearly half (1.69 ± 0.75 μmol O_2_ g^−1^ h^−1^) (*P* = 0.016). On air, oxygen uptake was lower but still higher after exposure to freshwater (1.29 ± 0.61 μmol O_2_ g^−1^ h^−1^) compared to that after exposure to 12 PSU (0.75 ± 0.18 μmol O_2_ g^−1^ h^−1^) (*P* = 0.015). The oxygen consumption rate in air, for all 20 snails before entering the salinity/medium exposure was on average 1.57 ± 0.67 μmol O_2_ g^−1^ h^−1^, with 1.38 ± 0.63 and 1.77 ± 0.68 μmol O_2_ g^−1^ h^−1^ for snails allocated to freshwater and brackish, respectively (*P* = 0.203). This value was not significantly different to the previous freshwater and post air freshwater respiration (*P* = 1).

#### Ammonium excretion rate

Like oxygen consumption, ammonium excretion rate was higher in freshwater (4.53 ± 1.81 μmol NH_4_^+^ g^−1^ h^−1^) than at 12-PSU salinity (2.132 ± 1.01 μmol g^−1^ h^−1^) (*P* = 0.003) (Fig. 4b).

#### Muscle water content

An acute exposure to brackish waters (12 PSU) caused a significant withdraw of intramuscular water, with a drop from 85.08 ± 2.11% in freshwater to 82.59 ± 2.27% at 12 PSU (Fig. 4c; *P* = 0.021).

## Discussion

This study explores the tolerance, survival, behaviour and physiology of the freshwater snail *C. dombeiana* after exposure to salinities higher than freshwater, through its whole ontogeny. Although tolerant to some degree, there was a clear detrimental effect of elevated environmental salinity on *C. dombeiana*, with the severity of these effects changing during its ontogeny. Embryonic development was completed up to salinities lower than 16 PSU, but with low survival. Adults, instead, tolerated up to 12 PSU after an acute 2.5 hours exposure and up to 8 PSU after 34 hours with no mortality.

*Chilina dombeiana* is oviparous with internal fertilization and direct development. The eggs are encapsulated and embedded in a gelatinous zigzag-like egg string, forming masses that are usually attached to rocky substrates (Bórquez *et al.*, 2015). In *C. dombeiana*, the direct and encapsulated development has been suggested to enable some tolerance to extreme environmental conditions (Bórquez *et al.*, 2015), yet our results show that embryos fail to develop optimally at salinities other than freshwater. The protective role of the egg mass has been shown after salinity exposure in *Melanochlamys diomedea*, with the embryos decreasing survival after passively loosing ~ 23% of their mass in low salinity in the absence of the egg mass (Woods and DeSilets, 1997). Egg capsules have been reported to further confer protection against desiccation and UV radiation in neogastropods (Rawlings, 1999). This further agrees with the hypothesis that for benthic marine invertebrate species that have mixed life histories, even a small period of encapsulation can significantly reduce mortality during mixed development (Pechenik, 1979). In the present study, salinity had detrimental effects on both embryonic survival and development even thought they were inside their protective egg mass. As eggs masses were cut in order to allocate similar maternal variance to each salinity treatment, it might also be possible that egg masses have lost their osmotic isolation, something that is worth testing in future experiments.

Embryonic development in *C. dombeiana* takes between 23 and 29 days at 18°C, presenting five stages with distinct characteristic features (Bórquez *et al.*, 2015). The duration of each stage in our experiment agrees with previous reports (Bórquez *et al.*, 2015). Salinity, however, had a significant effect on the duration of some stages. A significant difference was observed between 24 and 0 PSU in stage II, suggesting an immediate effect on the development of the trochophore larva stage, in which an elongated formation of the embryos is generated after the gastric phase. In fact, our results suggest that this initial process was disrupted at elevated salinity, as 100% mortality was registered in all replicates at 24 PSU in stage II. Although not properly quantified, dehydrated eggs and malformations were observed at 16 PSU. In other molluscs, such as the nudibranch *Rostanga arbutus*, both hyposaline and hypersaline (27 and 40 PSU) conditions increased the percentage of abnormalities on the developing embryos (Rose, 1986). Hatching also took longer than at lower salinities, suggesting developmental processes in previous stages were also affected.

In addition to growth, several organs must develop and processes must be completed such as the shell, digestive gland (stage III), torsion and formation of eye spots, generate cardiac activity (stage IV), shell calcification and complete foot and operculum formation (stage V). These stages are vulnerable to differences in osmotic pressures between the internal medium of the eggs and the external hypertonic environment. This could explain the malformations due to the plasmolysis and cell dehydration, likely given by the passive movement of intracellular water towards the outside (Entrekin *et al.*, 2019). As energy is allocated to maintain the internal environment, there will be a delay in growth and development while immersed in an external environment that results in an elevated diffusion gradient, as observed at high salinities. The impact of stress on metabolism and energy balance has also been studied in different animal models, leading to weight loss and a decrease in growth rates at salinities outside the species-specific optimal range (Mclusky, 1967; Steele and Steele, 1991; Boeuf and Payan, 2001).

Earlier stages of most animals have been traditionally considered more sensitive to changes in environmental salinity due to their greater surface area to volume ratios when compared to older stages (Kefford *et al.*, 2004). Kefford *et al.*, 2004, while assessing salinity tolerance in 12 taxa (encompassing groups of decapods, insects and snails, amongst others), found that salinity tolerance was greatly variable between eggs and hatchlings, ranging from 0.5 to 30.1 PSU, representing between 5% and 100% of the salt concentration that kills 50% of older stages (LC50) after 72 hours. This highlights potential overestimations that arise from assessing salinity tolerances using only adults, which agrees with our results. Time must also be taken into account because acute exposures could overestimate the long-term survival of adults. However, this does not apply to all species, as early stages and adults of six freshwater species (Mollusca, Arthropoda, Annelida) showed similar tolerances throughout the whole ontogeny (Kefford *et al.*, 2007). Based on these observations and our present work, we recommend the evaluation of salinity tolerance during the whole ontogeny of a species, to assess a more ‘functional salinity tolerance’.

### Adult preference and survival in aquatic and air medium

Pulmonata members are mostly tolerant to desiccation and other terrestrial environmental conditions (Naranjo-García and Olivera-Carrasco, 2014). Our results demonstrate that *C. dombeiana* mostly prefers the aquatic environment with respect to air, yet was able to uptake oxygen from both mediums. Freshwater gastropods capture oxygen from water or air; however, they probably differ in their ability to capture oxygen due to the intrinsic differences of water and air as respiratory mediums (Koopman *et al.*, 2016), and due to the differing efficiencies of the respiratory structures/epithelium used. Brace (1983) describes the precursor of a pulmonary plexus in *Chilina* spp., characteristic leading to the pulmonated group. He describes this enclosure of the mantle cavity, as an earlier pre-adaption of the mantle to function as a lung. Our results show that the preference for the aquatic environment matches their ability to respire in water better than in air. In this study, the snails were observed mostly submerged with a small number of emersions, even though they have the ability to emerse, and are frequently found emersed during low tide. The emersion behaviour was not even triggered when raising external salinity, as they still preferred to remain submerged in salty water instead of avoiding the hyperosmotic challenge by emersing. It is worth mentioning that animals subjected to tidal cycles might exhibit entrainment in some responses, and so it is advised to run treatment exposures in random order or in both directions (increasing and decreasing salinity) to avoid responses associated with potential biorhythms. We did not find any response during increasing salinity, and thus no response could be attributed to entrainment, but rather just a lack of avoidance response upon increasing salinity.

### Adult survival and physiological mechanisms

The different salinity treatments were shown to have a negative effect on the survival of snails, generating higher and faster mortalities as the salt concentration increased, when compared to freshwater (0 PSU). While it is not recommended to infer the sensitivity of species (SSD, species sensitivity distribution) between distant taxa, it may be reasonable to compare our results to related taxa (Kefford *et al.*, 2005). Exposure times (chronic or acute) are also crucial to consider amongst an array of different stressors (Urbina *et al.*, 2023). Exposure time represents 23.9% (Main Components Analysis, PCA) of the variance when including all data available for invertebrates (Cañedo-Argüelles *et al.*, 2012). When the authors separate the treatment (salinity) effects per period of exposure (before 24 hours and 72 hours of exposure), only the longest period registered a significant effect, with invertebrate density decreasing in all groups using a mesocosm approach (Cañedo-Argüelles *et al.*, 2012). Similar results were found in the density of invertebrates in cobblestones in another mesocosm exposure (Cañedo-Argüelles *et al.*, 2014). Thus, acute salinity exposures likely overestimate tolerances. In the case of our study, the exposure times were based on criteria such as tidal cycles and experimental survival, which are highly relevant factors.

When evaluating the relative tolerance to artificial seawater in macroinvertebrates of the Barwon River in Victoria, Australia, gastropods were one of the most sensitive groups. A study with the snail *Physa acuta* (Gastropoda: Physidae) found its LC50 at 72 hours to be 8.09 PSU, and while the species *Austropeplea lesonii*, *G. aliciae*, *Potamopyrgus antipodarum*, *A. tomentosa*, *G. gibbosa* are considered rare, the LC50 for these species varied between 5.03 and> 7.22 PSU. *Potamopyrgus antipodarum* belongs to the family Hydrobiidae, which has an operculum and presents greater tolerance (Kefford *et al.*, 2003). These salinity thresholds agree with those reported for *C. dombeiana* in this study, at ~ 8 PSU. On a mesocosm, Cañedo-Argüelles *et al.* (2014) reported significant changes in the abundance of different taxa (41) and thus community structure, where repeated short salinity pulses (lasting 3 hours) led to significant effect on the creek community (with a decrease in species richness and diversity due to the loss of the most sensitive taxa) at the lower salinity tested (2.68 PSU) after 72 hours (Cañedo-Argüelles *et al.*, 2012). Our results support the idea of delineating conservation efforts based on chronic responses to salinization, rather than acute.

All aerobic processes demanding energy should be included in the oxygen consumption rate. Oxygen consumption rates decreased during exposures to 12 PSU salinity compared to control freshwater. One explanation could be due lower cost of osmoregulation as snails approached their isosmotic point. The isosmotic point is when the osmotic pressures of the surrounding water and the internal osmotic pressure of the organism are the same (Brito *et al.*, 2000). However, if the decrease in oxygen consumption was due to this lower salt gradient, this should have been supported by no disturbances in percentage of water in muscular tissue. However, this was not the case, as a net and significant loss of intramuscular water was observed after exposure to 12 PSU. The more likely explanation would be the use of their operculum, as another behavioural response to isolate themselves from the external environment (Koopman *et al.*, 2016). This is likely to occur over the first 3.5 hours when oxygen consumption was measured, but unlikely to keep a tight seal over longer periods as it would demand anaerobic metabolism to be recruited. This explains the passive loss of foot muscle water found over 24 hours.

Operculum closure has been reported mainly in marine gastropods, yet *C. dombeiana* has an operculum despite being a freshwater species. Except for the Amphibolidae family, freshwater pulmonates lack an operculum in general. *Chilina dombeiana*, however, has a well-developed operculum (Bórquez *et al.*, 2015). On marine counterparts, gastropods such as *Littorina littorea, Purpura lapillus* and *Patella vulgata* quickly performed ‘carapace closure’ when directly transferred from seawater to 40% seawater. This quick response allowed these snails to only experience a transient hypoosmotic stress in the haemolymph and perivisceral fluids (Hoyaux and Jeuniaux, 1976). When *Stramonita brasiliensis,* a sea snail (35 PSU), is exposed to salinities ranging from freshwater to 70 PSU, it keeps their operculum open only between 20 and 55 PSU but closes it at salinities lower than 20 PSU and higher than 55 PSU (Veiga *et al.*, 2016). This operculum closure allows these snails to tolerate salinities above and below its optimum, like the strategy used for *C. dombeiana*, even when it could simply avoid elevated salinities by emersing. The maintenance of an operculum suggests not only that it evolved from marine ancestors, as previously suggested for most freshwater snails (Vermeij, 2000), but also that the species has only relatively recently colonized freshwater environments.

Snails exposed to 12 PSU also demonstrated lowered ammonium excretion rates compared to those exposed to 0 PSU (control), in agreement with our initial prediction. Pressley *et al.* (1981) showed the transfer of organisms to diluted media generates an increase in the active uptake of Na+ and a related increase in ammonium excretion. A decrease in ammonium excretion rate with increasing salinity has also been reported in other organisms (Lemos *et al.*, 2001; Re *et al.*, 2004; Tang *et al.*, 2005). When a freshwater organism is exposed to brackish water, the osmotic pressure of its blood is lower than that of the medium, facilitating passive movements of water away from the organism. If the animal can achieve and maintain homeostasis, intracellular water and ion content should experience only minor changes after changes in external salinity (Urbina *et al.*, 2013). A mechanism to decrease osmotic gradients, and therefore passive fluxes, is through the retention of ammonium (Parker & Haswell, 1987) or the release of amino acids to the haemolymph, thus decreasing the osmotic gradient. Both techniques may explain lower excretion rates at elevated salinities. Operculum closure would also result in a decreased flux of ammonium, and therefore rising ammonium levels inside snail tissues, but this remains to be confirmed. The observed decrease in muscular water content in the snails exposed at elevated salinity is also in agreement with previous studies in freshwater invertebrates (Horne, 1979; Veiga *et al.*, 2016). This decrease in intramuscular water content signals not only the passive movement of water from the intracellular compartments to the extracellular compartments but also the inability of *C. dombeiana* to regulate the osmolality of extracellular fluids in brackish waters. This water loss after 24 hours also means that operculum closure is not completely tight/efficient, and passive diffusion is decreased but not totally limited.

### Future perspective and salinization

Worldwide, climate change studies have mainly focused on its effect on temperature and acidification, with a small proportion of studies focusing on salinization, despite the projections of increased estuarine salinization worldwide. In Chile, such studies are scarce, and little we known about how estuarine salinization may impact freshwater diversity. Based on the results obtained, it is possible to conclude that *C. dombeiana* prefers the aquatic environment rather than remaining emersed even if salinity increase. However, it could uptake oxygen from both media. This certainly is advantageous, especially in fluctuating environments like estuaries, with tidal influence. The ability to remain emersed, in this snail, was shown only to be a short-term mechanism, with detrimental consequences in the long term.

Regardless of the ontogenetic stage, there is an inverse relationship between external salinity and survival. Although salinity has a slower effect on embryos, it is more critical than in adults, despite the presence of a gelatinous mass that should buffer them from the external environment. This research found that optimal embryonic development in salinities other than fresh water was not possible, but that adults were able to survive in brackish waters of 12 PSU, at least acutely. A follow-up study, comparing freshwater and estuarine populations of this snail, could help clarifying if the resistance to brackish water is a species attribute or only applies to the estuarine population used here. Also increasing the exposure time of adults to salinities up to 12 PSU will surely allow a better assessment of the impacts of salinization on this South American snail.

Estuaries are regulated by interactive physical processes including tides and river discharge (Robins *et al.*, 2016), making it hard to predict sea water intrusions. Our results show that freshwater salinization could generate negative impacts on invertebrate macrofauna, particularly for early stages of development. With low tolerance for salty waters, adults develop some degree of physiological tolerance to salinity, but this is aided by the behavioural response of isolating themselves by closing their operculum. Not only can climate change threaten to change the extent of sea water intrusion upstream, but anthropogenic pressure is also causing freshwater salinization, further exacerbating the need to evaluate the tolerance and effects of salinization in freshwater environments.

An ecological niche is the combination of environmental factors that are necessary and sufficient for the indefinite persistence of species (Hutchinson, 1957, 1978), and the incorporation of temperature and salinity tolerance limits have been proposed to better define the actual niche of a given species (Feng and Papes, 2017). Such approaches will likely facilitate improved predictions of future scenarios, and could support decision making and policies regarding conservation, especially when considering the endemism and status of an already vulnerable species.

## Funding

This work was supported by CONICYT-FONDECYT 1210071 to M.A.U. R.B. received financial support from ANID, ANID-PCHA/Doctorado Nacional/2019-21191604.

## Data Availability

The data shown in this article are available from the graphs, tables and on the online supplementary material. Yet, all data can be further shared on request to the corresponding author.
